# Trends in Ezetimibe Prescriptions as Monotherapy or Fixed-Dose Combination in Germany 2012–2021

**DOI:** 10.3389/fcvm.2022.912785

**Published:** 2022-06-13

**Authors:** Julius L. Katzmann, Marita Kieble, Salka Enners, Michael Böhm, Felix Mahfoud, Ulrich Laufs, Martin Schulz

**Affiliations:** ^1^Klinik und Poliklinik für Kardiologie, Universitätsklinikum Leipzig, Leipzig, Germany; ^2^Deutsches Arzneiprüfungsinstitut e.V. (DAPI), Berlin, Germany; ^3^Klinik für Innere Medizin III, Universitätsklinikum des Saarlandes, Homburg, Germany; ^4^Institut für Pharmazie, Freie Universität Berlin, Berlin, Germany

**Keywords:** lipid modifying agents, statins, ezetimibe, fixed-dose combination, drug utilization

## Abstract

**Aims:**

Addition of ezetimibe to statin therapy is recommended by current guidelines when low-density lipoprotein cholesterol (LDL-C) targets are not achieved with statin monotherapy. Fixed-dose combinations (FDC) improve medication adherence and facilitate risk factor control. We assessed prescription trends of ezetimibe as monotherapy or FDC with statins.

**Methods:**

Data from the *German Institute for Drug Use Evaluation* (DAPI) containing dispensing data of >80% of community pharmacies were analyzed. Prescriptions over time of lipid-lowering agents at the expense of the statutory health insurance (SHI) were extrapolated to all SHI-insured persons, representing approximately 88% of the total German population. Drug utilization was expressed as defined daily doses per 1,000 SHI-insured persons per day (DID).

**Results:**

Of all lipid-lowering drug prescriptions in 2021, 91.2% were statin monotherapy. Ezetimibe was prescribed as monotherapy or FDC with statin in 4.4 and 2.9%, respectively. DID steadily increased for statin (69%) and ezetimibe (424%) monotherapies between 2012 and 2021. In contrast, statin-ezetimibe FDC prescriptions exhibited only a minor increase (29%). The proportion of statin-ezetimibe FDC among all statin prescriptions was stable over time at approximately 3%. FDC prescription rates by specialists were higher compared to general practitioners and varied considerably between geographic areas.

**Conclusion:**

Combination lipid-lowering therapy is prescribed to a minority of patients. Prescriptions of ezetimibe as monotherapy increased to a much greater extent than statin-ezetimibe FDC. Considering the low proportion of patients achieving their LDL-C target and improved adherence to FDC compared to separate pills, statin-ezetimibe FDC may be utilized to improve the management of dyslipidemia.

## Introduction

Low-density lipoprotein cholesterol (LDL-C) treatment targets are only achieved in a minority of patients at elevated cardiovascular risk (1, 2). The ESC/EAS guidelines on dyslipidemia management recommend the addition of the cholesterol absorption inhibitor ezetimibe when the recommended LDL-C targets are not attained with lifestyle changes and statin medication (3). Current recommendations stress the need for early combination lipid-lowering therapy as first-line strategy and standard of care in secondary prevention patients (4, 5).

Ezetimibe is generically available in Germany since 2018. Fixed-dose combinations (FDC) of ezetimibe with several statins are available. In patients with arterial hypertension, FDC have been shown to improve medication adherence and blood pressure control (6, 7). In the 2018 ESC/ESH guidelines on hypertension, the initiation of antihypertensive treatment with FDC is recommended (class I) (8). Similarly, the use of statins and ezetimibe as FDC is associated with greater LDL-C reductions compared with prescription as separate pills (9).

Current prescription patterns of ezetimibe as monotherapy or FDC have not been reported yet. The data may provide important insights and opportunities to improve the management of dyslipidemia and attainment of LDL-C treatment targets. We analyzed the prescription trends and treatment cost of ezetimibe between 2012 and 2021 as monotherapy or FDC, calculated the proportion of statin-ezetimibe FDC prescriptions among all statin prescriptions, and compared this rate among different specialties and by geographic region using representative dispensing data from >80% of community pharmacies in Germany.

## Methods

This descriptive drug utilization study is based on data from the *German Institute for Drug Use Evaluation* (*Deutsches Arzneiprüfungsinstitut e.V.*, DAPI). The data were gathered from more than 80% (until June 2019) and more than 95% (from July 2019 onward) of all community pharmacies in Germany. The database contains anonymized dispensing data on medications prescribed at the expense of the statutory health insurance (SHI) funds. The available data were extrapolated by regional factors to represent all SHI-insured persons (approximately 88% of the total German population—that is, 73.3 million people) (6, 10). Prescriptions for privately insured patients are not covered, and no data on individual patients, treatment indication, duration, or dosages were available.

Using the specific product code (*Pharmazentralnummer*), dispensing data were linked to a database with information on the brand/generic name, composition, active ingredients, package size, dosage form, and administration route (11). All lipid-lowering agents (LLA) on the market were included in the analyses. Allocation of active ingredients was based on the official version of the German Anatomical Therapeutic Chemical (ATC) classification system with defined daily doses (DDD) published by the Federal Institute for Drugs and Medical Devices (12). The time course of dispensed LLA (ATC code C10) between 2012 and 2021 were analyzed per year in total and by drug classes with a focus on ezetimibe either as monotherapy prescription or as FDC with statins. The DDD per 1,000 SHI-insured persons per day (DID) were used as the unit of measurement as described previously (10). A direct age standardization was performed in order to control for the different age structures over time and between regions. The SHI population of Germany in 2021 served as the standard population.

The net cost per DDD was calculated as gross sales minus statutory manufacturer and pharmacy markdowns divided by the number of dispensed DDD. To explore the proportion of statin/ezetimibe FDC, the dispensings of all statin-containing products were defined as the denominator. This proportion was analyzed stratified by specialty of prescribing physician and by geographic region.

## Results

The overall prescriptions of LLA increased from 69.8 DID in 2012 to 118.2 DID in 2021. Among all LLA, statins represented the majority of prescriptions. In 2021, all statin prescriptions including FDC represented 94.2% and statin monotherapy 91.2%. The DID for statin-ezetimibe FDC and ezetimibe monotherapy were 3.5 and 5.2, respectively, representing 2.9 and 4.4% of all LLA prescriptions. Other LLA were only rarely prescribed [proprotein convertase subtilisin/kexin type 9 (PCSK9) inhibitors, inclisiran, or bempedoic acid] or with decreasing trends (fibrates): PCSK9 antibodies and inclisiran represented 0.25% and bempedoic acid 0.16% of all LLA prescriptions in 2021. Prescription of fibrates decreased from 2.1 DID in 2012 to 1.0 DID in 2021 with a proportion of 0.83% among all LLA in 2021. The data are shown in Table 1.

**TABLE 1:** Prescription of lipid-lowering agents 2012–2021 (in DID).

Year	Total	All statin prescriptions	Statin mono	Statin-ezetimibe FDC	Ezetimibe mono	Fibrate mono	PCSK9 inhibitor*	Bempedoic acid^#^	Other
2012	**69.8**	66.6	63.9	2.70	0.99	2.10	–	–	0.19
2013	**72.6**	69.6	67.3	2.34	0.94	1.91	–	–	0.09
2014	**74.3**	71.6	69.5	2.13	0.94	1.74	–	–	0.09
2015	**78.9**	76.1	73.9	2.24	1.08	1.63	0.00	–	0.09
2016	**83.6**	80.7	78.0	2.68	1.25	1.51	0.04	–	0.09
2017	**87.0**	83.9	80.8	3.03	1.49	1.37	0.09	–	0.10
2018	**91.6**	88.1	84.9	3.06	2.03	1.25	0.14	–	0.10
2019	**98.4**	94.2	90.8	3.25	2.68	1.16	0.18	–	0.10
2020	**107.2**	102.1	98.6	3.40	3.69	1.03	0.21	0.01	0.11
2021	**118.2**	111.4	107.8	3.47	5.19	0.98	0.29	0.19	0.11

*DID, defined daily doses per 1,000 statutory health insurance-insured persons per day; mono, monotherapy; FDC, fixed-dose combinations.*

*Includes proprotein convertase subtilisin/kexin type 9 (PCSK9) antibodies and inclisiran.

*^#^Includes monotherapy and bempedoic acid-ezetimibe FDC. Bold denotes the total values.*

**FIGURE 1:**
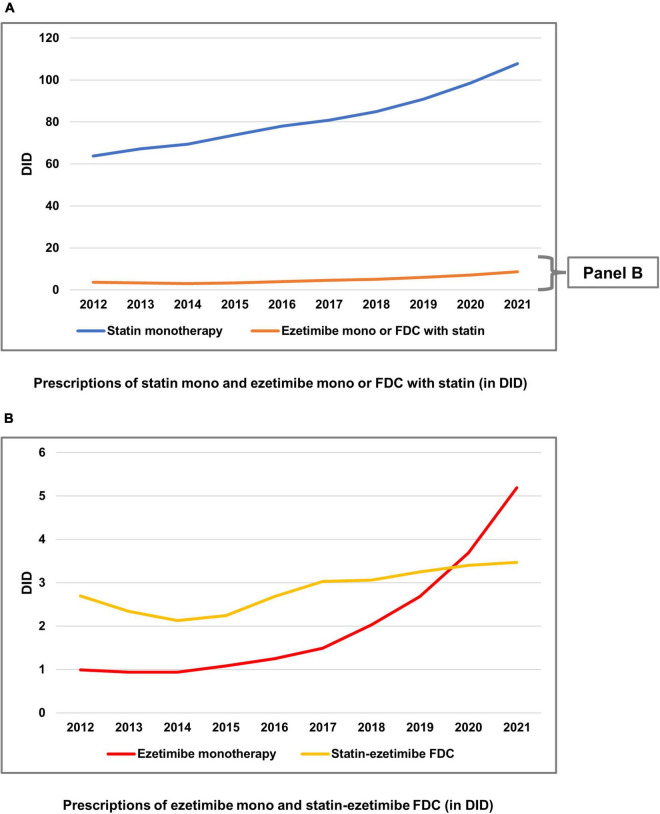
Prescriptions of statins and ezetimibe 2012–2021 in defined daily doses per 1,000 persons insured by statutory health insurance per day (DID). **(A)** Prescriptions of statin monotherapy and ezetimibe monotherapy or fixed-dose combinations (FDC) with statin (in DID). **(B)** Prescriptions of ezetimibe monotherapy and statin-ezetimibe FDC (in DID). DID, defined daily doses per 1,000 statutory health insurance-insured persons per day; FDC, fixed-dose combinations; mono: monotherapy.

Figure 1A shows a steady increase 2012–2021 in statin monotherapy and ezetimibe prescriptions including ezetimibe monotherapy and statin-ezetimibe FDC. In this period, statin DID increased by 69%. The development in ezetimibe prescriptions divided in monotherapy and statin-ezetimibe FDC is shown in Figure 1B. While there was a steep increase in ezetimibe monotherapy prescriptions (increase 2012–2021: 424%), the prescriptions of statin-ezetimibe FDC decreased between 2012 and 2014 and only slightly increased afterward (increase 2012–2021: 29%).

**FIGURE 2:**
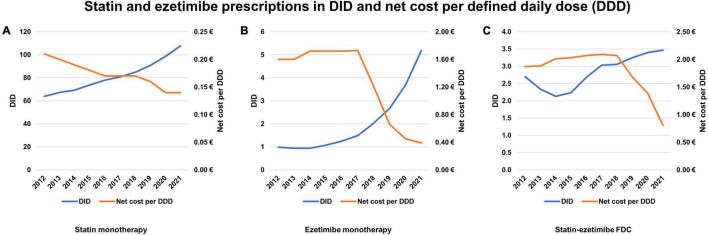
Prescriptions of statins and ezetimibe 2012–2021 in defined daily doses per 1,000 persons insured by statutory health insurance per day (DID) and net cost per defined daily dose (DDD). **(A)** Statin monotherapy. **(B)** Ezetimibe monotherapy. **(C)** Statin-ezetimibe FDC. DDD, defined daily dose; FDC, fixed-dose combinations; DID, defined daily doses per 1,000 statutory health insurance-insured persons per day.

The developments in prescriptions and net cost per DDD are depicted in Figure 2. Figure 2A demonstrates a steady decrease in statin net cost per DDD, while DID steadily increased. Figure 2B shows a significant drop in net cost per DDD for ezetimibe monotherapy from 2018 onward, coinciding with the availability of generic ezetimibe in Germany. This was accompanied by a steep increase in DID. Finally, Figure 2C shows a similar pattern for statin-ezetimibe FDC, however the drop in net cost per DDD occurred delayed in 2019. The DID of FDC increased by a smaller extent than those of ezetimibe monotherapy.

**FIGURE 3:**
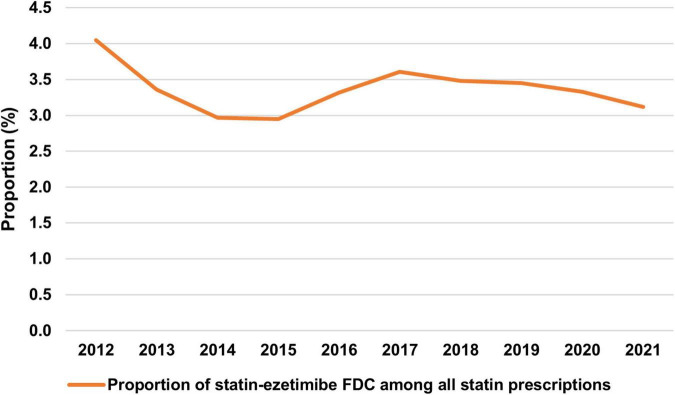
Proportion of statin-ezetimibe FDC among all statin prescriptions. FDC, fixed-dose combinations.

The proportion of statin-ezetimibe FDC among all statin prescriptions ranged at approximately 3% with no clear trend between 2012 and 2021 (Figure 3).

The majority of all statin prescriptions were carried out by general practitioners (95.1% in 2021). The proportion of statin-ezetimibe FDC prescriptions among all statin prescriptions was higher for specialists including cardiologists, internal specialists, and nephrologists, ranging from 5.2 to 7.1% as compared to 3.0% for general practitioners (Table 2).

**TABLE 2:** Prescription of statins in DID and proportion of statin-ezetimibe FDC by specialty 2021.

Specialty	DID	Proportion of statin prescriptions by specialty according to DID (%)	Proportion of statin-ezetimibe FDC among all statin prescriptions (%)
**Total**	**111.4**	**100.0**	**3.1**
General practitioners	106.0	95.1	3.0
Cardiologists	1.8	1.6	7.1
Internal specialists	1.1	1.0	5.2
Nephrologists	1.0	0.9	5.7
Others	1.6	1.4	4.5

*DID, defined daily doses per 1,000 statutory health insurance-insured persons per day; FDC, fixed-dose combinations. Bold denotes the total values.*

The age-standardized prescriptions of statins were comparable in different geographic regions—that is, the 16 federal states in Germany. However, the proportion of statin-ezetimibe FDC among all statin prescriptions varied considerably from 1.7 to 7.0% (Table 3).

**TABLE 3:** Prescription of statins in DID and proportion of statin-ezetimibe FDC 2021 by geographic region (16 federal states).

Region/State	Statins in DID	Proportion of statin-ezetimibe FDC among all statin prescriptions (%)
Saxony-Anhalt	120.5	7.0
Mecklenburg West Pomerania	120.6	6.1
Saxony	104.2	5.3
Brandenburg	124.8	5.1
Thuringia	118.0	4.3
Saarland	136.9	3.6
Hesse	99.7	3.2
**Germany**	**111.4**	**3.1**
Bavaria	113.7	3.0
Baden-Wuerttemberg	105.9	3.0
Lower Saxony	102.8	2.8
Berlin	126.4	2.8
Hamburg	109.4	2.4
Rhineland-Palatinate	119.7	2.0
Westphalia-Lippe	111.5	1.9
Schleswig-Holstein	108.1	1.8
North Rhine	112.3	1.8
Bremen	108.0	1.7

*DID, defined daily doses per 1,000 statutory health insurance-insured persons per day; FDC, fixed-dose combinations. Bold denotes the total values.*

## Discussion

The main findings of this study are that (1) combination lipid-lowering therapy is only prescribed to a minority of patients, (2) the increase in ezetimibe prescriptions is primarily due to monotherapy rather than statin-ezetimibe FDC, and (3) the rates of statin-ezetimibe FDC prescriptions among all statin prescriptions vary considerably among different specialties and by geographic region. Our findings contribute to the understanding of the low proportion of patients achieving their LDL-C target and show potential for improvement of lipid management in cardiovascular prevention.

### Low Proportion of Lipid-Lowering Agents Combination Therapies

Among all LLA prescriptions, statin monotherapy represented 91.2% in 2021. Although it is likely that some patients with statin monotherapy concomitantly received ezetimibe as a separate pill, even in the theoretic assumption of all ezetimibe monotherapy prescriptions being prescribed in addition to statin monotherapy, still the vast majority would have received statin monotherapy. This finding provides at least a part of the explanation for the low proportion of patients achieving their LDL-C target. At the same time, this observation opens the opportunity to improve patient care by enhancing the utilization of LLA (fixed-dose) combination therapy.

In the IMPROVE-IT study, the addition of ezetimibe to simvastatin in patients after acute coronary syndrome reduced LDL-C to a median of 54 mg/dL, which corresponds to the current LDL-C treatment target in patients at very high risk (13). A simulation study using an US administrative database concluded that an LDL-C target of <70 mg/dL could be achieved in 99.3% of patients, while 67% would require statin monotherapy, 19% the addition of ezetimibe, and 14% a PCSK9 inhibitor (14). A Swedish simulation study suggested that 49.3% of patients with myocardial infarction could attain the LDL-C treatment target of <55 mg/dL with intensified statin therapy and ezetimibe (15). In a German simulation study, it has been suggested that 42% of patients with atherosclerotic cardiovascular disease would require a PCSK9 inhibitor to achieve the LDL-C target of < 55 mg/dL, which could be attained in 97.9% (16). These studies demonstrate a large gap between the available treatment options and their clinical implementation. While the prescription of PCSK9 antibodies as the third cornerstone in LDL-C lowering therapies (3) is limited by high treatment costs and restrictions for reimbursement, our study underlines that the consequent utilization of the generically available combinations of statin and ezetimibe as the second treatment step still has potential for improvement: Despite the large relative increases in ezetimibe prescriptions from 2018 onward, the proportion of ezetimibe prescriptions in comparison to statin prescriptions remains small.

### Increase in Ezetimibe Monotherapy Prescriptions

From 2012 to 2021, ezetimibe monotherapy prescriptions increased fivefold. This increase was most pronounced from 2018 onward and is likely related to the reduction in costs associated with the generic availability of ezetimibe in 2018 and the release of the ESC/EAS guidelines on dyslipidemia in 2019 (3). In contrast, statin-ezetimibe FDC prescriptions increased only slightly. Based on our data, it is not possible to distinguish which proportion of patients with ezetimibe monotherapy received only ezetimibe or concomitantly received a statin, i.e., combination statin-ezetimibe therapy with separate pills as alternative to an FDC. In a previous study, in 2018, approximately 92% of patients who were treated with both a statin and ezetimibe, received an FDC and 8% separate pills (9). As the generic availability of ezetimibe in this year may have had an impact on the prescription behavior, and the FDC cost decreased with delay, it can only be speculated how the proportion of separate pills vs. FDC would have developed without this confounder. However, the data suggest that prize is not the only reason for the low number of patients on FDC.

Nonetheless, both possible scenarios offer opportunities for improvement: If the large increase in ezetimibe monotherapy prescriptions was due to ezetimibe monotherapy without statin, this would underscore the importance of attempts to establish statin therapy in patients without statin therapy. Reasons for not prescribing or discontinuing statin therapy include side effects such as an increased risk of new-onset diabetes and a raise in liver transaminases (17), however there are only few contraindications for statin therapy. Importantly, many patients struggle with statin-associated muscle symptoms. In a recent meta-analysis including more than 4 million patients, the overall prevalence of statin intolerance was 9.1% and might often be overestimated (18). N-of-1 trials such as the SAMSON study demonstrated that also in patients with a history of statin intolerance, effective statin therapy could be established in approximately 50% of patients with prior symptoms (19).

On the other hand, if the increase in ezetimibe monotherapy mainly represented an increase in prescriptions of statins and ezetimibe as separate pills, this would open the opportunity to improve care by enhancing the utilization of FDC. For antihypertensive and lipid-lowering treatment, FDC compared to separate pills lead to improved medication adherence, improved persistence, better control of blood pressure and LDL-C concentrations, respectively (7, 20), and are associated with a lower incidence of cardiovascular events and lower all-cause mortality (21). Improved medication adherence has been associated with favorable outcomes (22–24). The 2018 ESC/ESH guidelines on hypertension recommended FDC treatment rather than separate pills in most patients (8). In contrast, there is no such recommendation in the current ESC/EAS dyslipidemia guidelines (3). Randomized trials on FDC vs. separate pills in lipid-lowering treatment are lacking. Retrospective studies from Australia did not find an improvement in adherence comparing statin/ezetimibe FDC vs. separate pills (25, 26). However, beneficial effects of statin-ezetimibe FDC on adherence still appear likely, considering the evidence for FDC antihypertensives and the fact that adherence to blood pressure- and lipid-lowering medication strongly correlates (27). Furthermore, a previous observational, longitudinal study showed more profound LDL-C reductions after prescription of statin-ezetimibe FDC as compared to separate pills (9), which underscores the concept of primarily treating patients with FDC rather than separate pills. Given the same drug costs (Figure 2) for statin-ezetimibe FDC and separate pills, there is virtually no argument for not prescribing FDC.

### Different Rates of Statin-Ezetimibe Fixed-Dose Combinations Prescriptions Across Specialties and Geographic Region

Finally, there were remarkable differences in the rates of FDC prescription as proportion of all statin prescriptions among the different prescriber specialties and by geographic region. The rate of FDC prescriptions was lowest among general practitioners. Part of the explanation for this finding may be systematic differences in the cardiovascular risk of patients treated by each specialty and a higher awareness in specialists preferably treating patients with elevated risk. Given the fact that general practitioners were responsible for 95.1% of all statin prescriptions, higher rates of FDC prescriptions among general practitioners would have a great impact on the total FDC prescriptions. The geographic differences related to FDC prescriptions cannot be fully explained based on the available data. In the federal states of former East Germany (Saxony-Anhalt, Mecklenburg-Western Pomerania, Saxony, Brandenburg, and Thuringia), the FDC rates were higher compared to those in West Germany. Reasons may include local differences in reimbursement, structure of healthcare in cities vs. rural areas, and population-related differences. Further research is required to identify opportunities for improvement especially in the areas with the lowest rates of FDC prescription.

### Strengths and Limitations

A strength of this study is that it is based on data obtained from the vast majority (since July 2019 > 95%) of community pharmacies in Germany and, therefore, covers a large part of the German population. A limitation is that concomitant therapies including other LLA are not available in the data set. Especially, it is not possible to distinguish which proportion of patients with ezetimibe monotherapy received only ezetimibe or concomitantly received a statin. Furthermore, there were no data on laboratory markers, patient characteristics including comorbidities, medication adherence, or dosages.

## Conclusion

In Germany, combination lipid-lowering therapy is only prescribed to a minority of patients. Given the low proportion of patients achieving their LDL-C target on the one hand and studies providing evidence that most patients can achieve even ambitious LDL-C targets on the other hand, further utilization of combining LLA appears promising to improve LDL-C target attainment. To accomplish long-term medication adherence, the primary prescription of FDC rather than separate pills provides an opportunity to improve care, especially considering that the current trends observed in this study suggest that FDC are not preferably prescribed.

## Data Availability Statement

The data underlying this article will be shared on reasonable request to MS.

## Author Contributions

JK, MK, SE, UL, and MS contributed to the conception and design of the work. MK and SE contributed to the acquisition and analysis of the data for the work. JK, MK, SE, and MS drafted the manuscript. All authors contributed to the interpretation of the data, critically revised the manuscript for important intellectual content, gave final approval, and agreed to be accountable for all aspects of the work.

## Conflict of Interest

MB was supported by the Deutsche Forschungsgemeinschaft (German Research Foundation; TTR 219, project number 322900939), reports personal fees from Abbott, Amgen, Astra Zeneca, Bayer, Boehringer Ingelheim, Bristol Myers Squibb, Cytokinetics, Medtronic, Novartis, Servier, and Vifor, and participation in advisory boards for Amgen, Bayer, Boehringer Ingelheim, Cytokinetics, Medtronic, Novartis, Pfizer, ReCor, Servier, and Vifor, all outside the submitted work. FM was supported by Deutsche Gesellschaft für Kardiologie (DGK), Deutsche Forschungsgemeinschaft (SFB TRR219), Deutsche Herzstiftung, Medtronic, and Recor Medical and has received scientific support and/or speaker honoraria from AstraZeneca, Bayer, Boehringer Ingelheim, Medtronic, Merck, and ReCor Medical, all outside the submitted work. UL has received speaker honoraria from Amgen, Daiichi Sankyo, Novartis, and Sanofi. MS has received speaker honoraria from BMS, DGK-Academy, and Pfizer and consulting fees from MSD and Vifor, all outside the submitted work. The remaining authors declare that the research was conducted in the absence of any commercial or financial relationships that could be construed as a potential conflict of interest.

## Publisher’s Note

All claims expressed in this article are solely those of the authors and do not necessarily represent those of their affiliated organizations, or those of the publisher, the editors and the reviewers. Any product that may be evaluated in this article, or claim that may be made by its manufacturer, is not guaranteed or endorsed by the publisher.
